# Chemical Versus Biological Approaches to the Synthesis of Lactobionic Acid: A Review

**DOI:** 10.3390/molecules30163330

**Published:** 2025-08-09

**Authors:** Wiktoria Piątek-Gołda, Monika Osińska-Jaroszuk, Anna Pawlik, Elwira Komoń-Janczara, Justyna Sulej

**Affiliations:** 1 Department of Biochemistry and Biotechnology, Institute of Biological Sciences, Maria Curie-Sklodowska University, 19 Akademicka St., 20-033 Lublin, Poland; wiktoria.piatek.golda@gmail.com (W.P.-G.); monika.osinska-jaroszuk@mail.umcs.pl (M.O.-J.); anna.pawlik@mail.umcs.pl (A.P.); 2 Department of Biotechnology, Microbiology and Human Nutrition, University of Life Sciences in Lublin, 8 Skromna St., 20-704 Lublin, Poland; elwira.komon.janczara@up.lublin.pl

**Keywords:** lactobionic acid, production, chemical methods, biological methods

## Abstract

Lactobionic acid, widely used in the pharmaceutical, cosmetic, and food industries, is produced through chemical and biological methods, each with distinct advantages and challenges. This review examines the key approaches to its production, highlighting the chemical oxidation of lactose and biotechnological processes using microbial and enzymatic systems. Chemical methods offer high yields and rapid production but are often hindered by environmental concerns and lower product purity. In contrast, biological methods provide eco-friendly alternatives with superior product quality, albeit with limitations in scalability and higher initial costs. The comparison in this overview focuses on productivity, cost efficiency, purity, and environmental impacts, underscoring the potential of biological methods to align with sustainability goals. This review highlights further advances in biological approaches and explores hybrid solutions that combine the strengths of both methods for optimal lactobionic acid production.

## 1. Introduction

**Figure 1:**
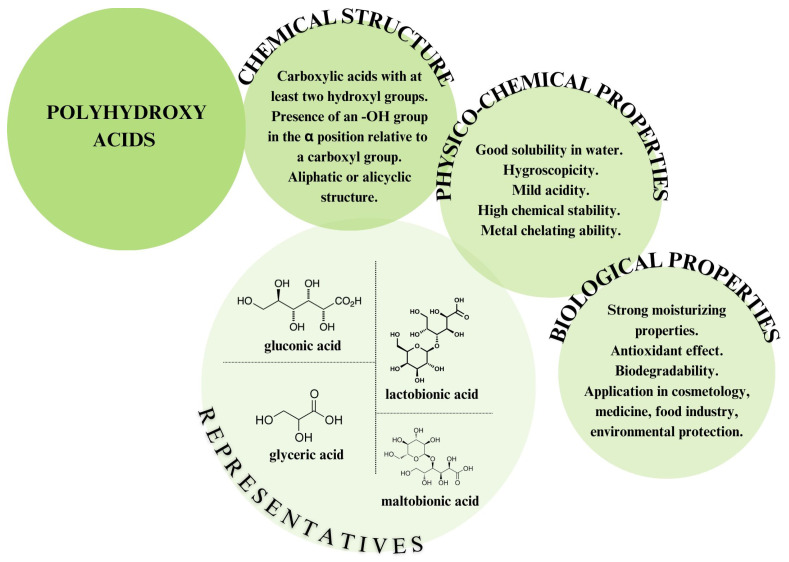
Schematic overview of polyhydroxy acids (PHAs).

Since the late 1940s, hydroxy acids, which include alpha-hydroxy acids (AHAs), beta-hydroxy acids (BHAs), and the more recently developed polyhydroxy acids (PHAs), have been chemically synthesised for industrial applications [1,2]. PHAs are a class of organic carboxylic acids characterised by the presence of two or more hydroxyl groups attached to the carbon atoms of an aliphatic or alicyclic chain (Figure 1) [3]. Each PHA molecule contains a single carboxyl group that gives it acidic properties, while the other hydroxyl groups have a neutral net charge. A characteristic feature in the structure of PHAs is that at least one hydroxyl group must be in an alpha position relative to the carboxyl group [1].

PHAs represent a new generation of hydroxy acids that also share the beneficial properties of AHAs but have some additional advantages [4]. Their larger molecular size contributes to their gentler effects on the skin and significantly reduces the risk of side effects. PHAs penetrate the skin more slowly and act more superficially, decreasing the likelihood of irritation or redness. All of this makes polyhydroxy acids suitable for people with sensitive skin, vascular skin, and even rosacea [3,5]. PHAs exert exfoliating, moisturising, and antioxidant effects [6]. This makes them especially attractive in the cosmetics industry [4,7]. Besides personal care products, PHAs also have potential for applications in pharmaceuticals, medicine, and the food industry [4,8]. However, the use of PHAs in the food industry is still limited owing to a deficiency of research on PHAs in this sector and a lack of a regulatory framework for their widespread usage [9,10]. 

**Figure 2:**
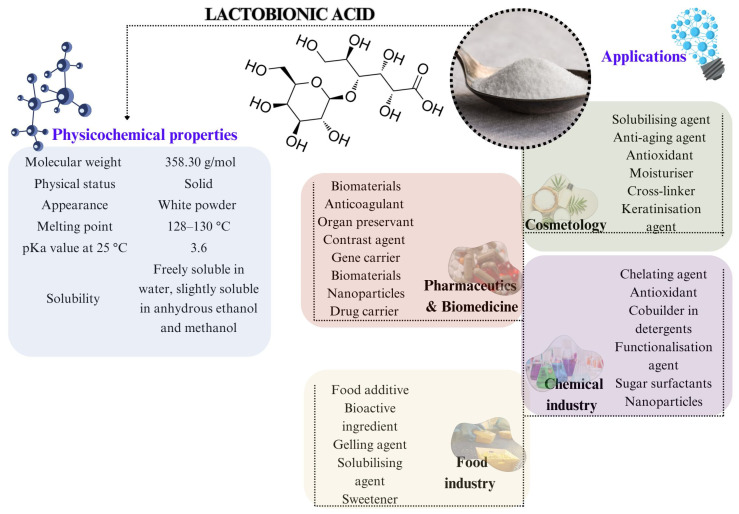
Summary of the physicochemical properties and applications of lactobionic acid.

Among PHAs, lactobionic acid (LBA) is one of the most popular polyhydroxy bionic acids in biotechnology. This lactose derivative with the chemical formula of 4-O-β-D-galactopyranosyl-D-gluconic acid and the molecular formula C_12_H_22_O_12_ is formed via the partial oxidation of the lactose glucose molecule into gluconic acid (Figure 2) [11,12]. It has a molecular weight of 358.30 and a pKa value of 3.6 at 25 °C [11,13]. LBA is most commonly a pale white hygroscopic powder which readily dissolves in water but has a relatively low solubility in anhydrous alcohols, including methanol and ethanol [2].

Structurally, lactobionic acid is classified as an oligoaldonic acid containing galactose bound to gluconic acid through an ether-like bond [4]. Its hygroscopic behaviour is, in part, due to the presence of eight hydroxyl groups of the molecule. LBA belongs to the same group of molecules with prebiotic properties, such as lactulose and galacto-oligosaccharides [14]. The health benefits of LBA have been attributed to its antioxidant, antimicrobial, chelating, stabilising, acidifying, and hydrating properties [10]. The interest in lactobionic acid has increased in the food industry, although the US Food and Drug Administration (FDA) and the European Union do not currently approve the use of LBA in food, unlike lactobionate salts. Nevertheless, it holds promise as a food additive serving as a calcium carrier, acidity regulator, or antioxidant [10,15]. This bio-based product could be of great value to the dairy industry due to its lactose-derivative nature [16].

In recent years, the increasing commercial significance of lactobionic acid has greatly influenced the intensification of work aimed at creating biotechnological systems for its production [10,11]. These approaches are a promising alternative to conventional chemical synthesis, which is by far the most commonly used in industry [10]. This review discusses in detail the latest comparison of chemical and biotechnological lactobionic acid production routes, encompassing their respective advantages and limitations.

## 2. Development of Lactobionic Acid Production Methods

**Figure 3:**
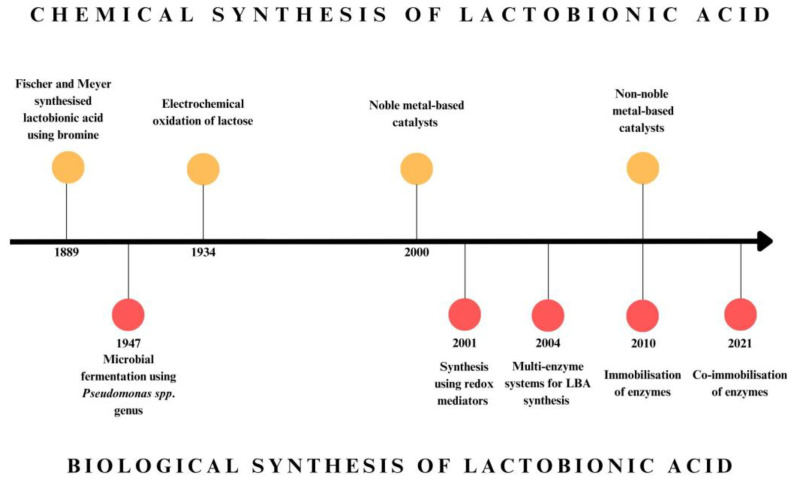
Timeline of key discoveries (milestones) in lactobionic acid production methods, including chemical methods (orange markers) and biological methods (red markers).

The production of lactobionic acid involves the selective conversion of lactose by oxidising the free aldehyde group of glucose to a carboxyl group. Several techniques, including heterogeneous catalytic oxidation, electrochemical oxidation, or biocatalytic production utilising enzymes or microorganisms, have been investigated to produce LBA [10]. In industrial applications, LBA is usually produced via chemical synthesis; however, recent research has highlighted bioconversion methods due to their improved selectivity, specificity, and the use of milder conditions [17]. In 1889, Fischer and Meyer chemically synthesised lactobionic acid for the first time, using bromine water in the process [18]. Initially, the chemical methods for synthesising LBA relied on stoichiometric oxidation of lactose, utilising such chemicals as the aforementioned bromine or iodine as catalysts [19]. Investigation of approaches for the chemical synthesis of lactobionic acid focused on two methods: heterogeneous oxidation and electrochemical oxidation. Electrochemical oxidation techniques have long been employed for the synthesis of lactobionic acid (LBA). An early example is Isbell’s 1934 work, which involved the production of calcium lactobionate [15,20]. Since 2000, research efforts have concentrated on the application of noble metals as catalysts. Although there are many patents and publications, noble metal-based catalysts for sugar oxidation in the presence of oxygen are a concept that is very difficult to scale up due to the cost of the process [21]. A brief timeline of progress in lactobionic acid production methods is provided in Figure 3.

The progress in chemical methods involves the utilisation of metallic catalysts, including non-noble metals or noble metals with promoters. An example of such work is the study of palladium (Pd) catalysts promoted by bismuth (Bi) [22]. Another studied area is the catalytic wet oxidation of lactose using non-noble metal oxides, such as manganese (Mn) and cerium (Ce) [23]. The research endeavours in the chemical synthesis of lactobionic acid are characterised by an intensive search for efficient and selective catalysts with an emphasis on minimising the negative environmental impact and improving the economic viability of the process compared to traditional methods. Despite their efficacy, these techniques presented significant dangers to the environment and public health since they required extensive purification and produced hazardous by-products. Catalytic systems that provide higher efficiency and align with the tenets of green biotechnology have drawn so much interest.

In contrast to chemical production methods, which had been around for a long time, biological methods were discovered in the 1940s. Stodola and Lockwood described the possibility of producing aldobionic acids using *Pseudomonas* species [24]. This Gram-negative bacterium is among the earliest and most extensively researched organisms that produce lactobionic acid. The initial microorganism recognised for its ability to produce LBA was *Pseudomonas taetrolens*, formerly referred to as *Pseudomonas graveolens*. Since then, many groups of microorganisms have been studied for LBA production. In addition to these bacteria, the genera *Burkholderia*, *Zymomonas*, and *Acetobacter* are also undergoing rigorous investigation and application for the bioproduction of LBA. Additionally, several fungal species exhibit the capability to generate LBA or contain enzymes applicable in this process, and red algae constitute a somewhat obscure yet promising category of LBA producers [10,15,25].

The “green” trend in LBA production entails the use of isolated enzymes with the ability to oxidise lactose. These methods hold promise due to the high selectivity of biocatalysts, which simplifies the LBA purification process after the reaction, a crucial aspect for industrial applications, especially in the pharmaceutical and food sectors [15]. The development of enzymatic methods for lactobionic acid production focuses on several key areas aimed at increasing process efficiency and enzyme stability. Although the first report of lactose conversion to LBA using cellobiose dehydrogenase (CDH) dates to 1982 [26], the research on the use of redox mediator systems in LBA production, which began in the early 21st century, was a milestone. The application of mediators, i.e., molecules characterised by reversible redox transitions and appropriate potentials that enable efficient electron transfer from the active centre of the enzyme to an electrode or another acceptor, has led to the creation of multi-enzymatic systems. The research conducted by Baminger et al. (2001) was the first example of a system consisting of an enzyme (CDH), a redox mediator, and a regenerating enzyme (laccase) applied in LBA production [27].

Further research on the use of enzymes in LBA biosynthesis primarily involves the immobilisation and co-immobilisation of multi-enzymatic systems on various carriers, such as chitosan or porous silica. This approach creates cascade systems combining biocatalysts with complementary functions, such as the already mentioned CDH and laccase, which cooperate in the oxidation of lactose to LBA [28,29].

## 3. Chemical Methods of Lactobionic Acid Production

The selective oxidation of disaccharides poses a significant challenge because these molecules possess multiple carbons susceptible to oxidation. Developing a catalyst capable of differentiating the anomeric hydroxyl (C1) from the two primary alcoholic groups (C6, C6′) is particularly problematic [30]. Catalytic oxidation of lactose typically follows specific pathways depending on the catalyst system employed. The transformation involves oxidation of the aldehyde group at the C1 position of the glucose moiety in lactose to form a carboxylic acid group, resulting in lactobionic acid [15]. In the oxidation of disaccharides, Pd, Pt, and Au with different conformations (monometallic, bimetallic, and bifunctional catalysts and the use of the metallic promoter) are some of the most extensively utilised selective chemical catalysts [30,31]. The choice of support for solid catalysts is essential, as they function as catalytically active centres, enhancing the efficiency of the supported metals or metal oxides [32]. In addition to selecting the appropriate catalyst, one of the most important steps in catalytic methods is the choice of oxidants. This is a key factor, as it determines the reactant’s durability and performance. Conventional oxidisers, such as bromine and nitric acid, have been replaced by mostly environmentally friendly alternatives. Molecular oxygen (O_2_) and hydrogen peroxide (H_2_O_2_) are considered green oxidisers because they leave harmless, inexpensive, and abundant by-products after degradation [33,34,35].

Chemical methods for producing lactobionic acid have made tremendous progress due to improvements in catalytic systems, greener oxidants, and more optimal process conditions. While these traditional one-to-one stoichiometric oxidation methods of small-scale synthesis are still relevant, the synthesis of lactobionic acid via catalytic approaches involving noble metals along with green oxidants may represent the next generation of industrial lactobionic acid production. However, the application of green oxidants like O_2_ and H_2_O_2_ does not totally eliminate pollution concerns. There are still environmental dangers associated with the use of metallic catalysts because of the process energy consumption or possible wastewater pollution.

Therefore, it was necessary to develop new systems for biotechnological LBA production that are sustainable, environmentally friendly, and efficient.

This section summarises the main chemical methods, reactions, and processing conditions available for lactobionic acid, including the latest information on catalytic systems and processing optimisation.

### 3.1. Heterogeneous Catalytic Oxidation

A process known as heterogeneous catalysis occurs when the catalyst is in a different phase than the reactants; typically, the catalyst is in a solid phase while the reactant is in a liquid or gaseous phase [36,37]. Researchers are constantly creating better catalysts with increased activity and selectivity as the field of heterogeneous catalytic oxidation continues to advance. For industrial applications, transitioning from homogeneous to heterogeneous catalytic systems offers several benefits, such as simpler catalyst recovery and reuse [36,38]. Despite the limitations, this process is still a promising area of research due to its potential integration with existing lactose transformation technologies [15]. In addition, the advantages of using heterogeneous catalysts include their easy post-reaction recovery, recycling potential, and relatively lower cost compared to homogeneous catalysts and biocatalysts [39].

**Figure 4:**
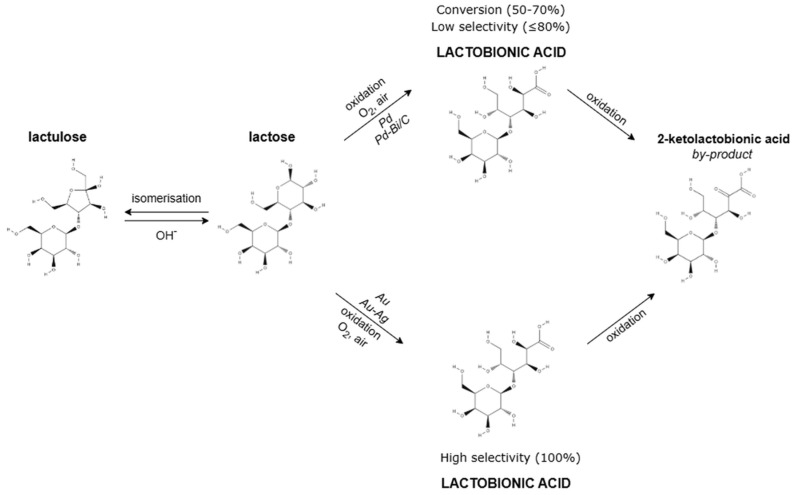
Scheme of heterogeneous catalytic oxidation of lactose to lactobionic acid in the presence of various metallic catalysts in alkaline conditions.

Depending on the catalyst’s activity and selectivity as well as the conditions of the reaction (pH, temperature, pressure, O_2_ concentration), the heterogeneous catalytic lactose oxidation process may result in the production of undesired by-products, such as lactulose (isomerisation to lactulose is highly preferred at pH values between 8.0 and 9.0) and keto acids. A schematic diagram of the heterogeneous catalytic oxidation of lactose to lactobionic acid and reaction by-products is shown below (Figure 4).

The synthesis of lactobionic acid through heterogeneous catalytic processes using air or molecular oxygen as oxidising agents is considered an environmentally sustainable alternative to the use of biological methods, offering faster conversions and reduced effluents and by-products compared to biocatalytic reactions [39].

In studies on the selective synthesis of lactobionic acid through heterogeneous oxidation of lactose, a wide range of monometallic (Ru, Ni, Pd, Pt, Au), bimetallic (Pd-Bi, Pt-Bi, Au-Pd, Au-Ag), and even trimetallic (Pd-Pt-Bi) catalysts on various supports (activated carbon, Al_2_O_3_, SiO_2_, TiO_2_, CeO_2_) were investigated. The two-step oxidation of lactose to LBA using Pd and Pd-Bi/C catalysts at pH 7 can lead to the formation of an undesirable by-product, i.e., 2-ketolactobionic acid. Low selectivity (≤80%) and limited conversion (50–70%) are common in this reaction [40,41]. In contrast, Au or Au-Ag catalysts supported on Al_2_O_3_, SiO_2_, TiO_2_, and CeO_2_ exhibit nearly 100% selectivity toward lactobionic acid at 65 °C and pH 9, with the highest lactose conversion rates. Conversely, the second step, where lactobionic acid is converted to 2-ketolactobionic acid, is often less selective [42,43].

Catalysts based on noble metals, such as platinum (Pt), palladium (Pd), and ruthenium (Ru), have been widely studied [44,45]. In the early 21st century, research was undertaken on the selective oxidation of lactose with Au/TiO_2_ and Au/Al_2_O_3_ catalysts, which achieved 100% selectivity for lactobionic acid [30]. Research conducted by Vlad-Cristea suggests that Bi-Pt/C catalysts converted lactose to lactobionic acid in a highly efficient manner [46]. Likewise, at very low concentrations of molecular oxygen (O_2_), Pd-Bi catalysts demonstrated a 96% conversion yield with 100% selectivity toward lactobionic acid formation [47]. Furthermore, the use of gold nanoparticles that are supported on materials like titanium dioxide (TiO_2_) or zeolites usually have very high catalytic performances and reuses, which are appealing for industrial applications because of their selective oxidations involving no or minimal side reactions [15]. Several catalyst systems have been reported in the literature (Table 1).

### 3.2. Electrochemical Oxidation

Electrochemistry has recently regained prominence as a clean, carbon dioxide emission-neutral method to drive chemical transformations. In chemical synthesis, electrochemistry eliminates the need for chemical oxidising/reducing agents and ensures safer processing conditions by separating reactions into half-cell processes [56].

The electrocatalytic oxidation of lactose is another chemical method used in the synthesis of lactobionic acid [57]. Electrocatalysis constitutes a significant field of electrochemistry, where principles of heterogeneous catalysis apply within interfacial electric fields. In such systems, the rate of electrochemical reactions is typically enhanced without consuming or altering the electrocatalyst [58]. By applying an external potential, the current passes through an electrolyte solution or molten electrolyte, inducing substrate oxidation in the electrolyte at the anode and reduction reactions at the cathode [59].

The catalyst type plays a critical role in electrocatalysis, accelerating reactions without being consumed, making these materials essential components across electrochemical systems. Leveraging electrocatalytic technology principles, substrates, including various organic and inorganic substances, can be dissolved in electrolyte solutions and electrolytically catalysed to produce target oxidation or reduction products using only water as the oxidant or reductant [59]. The interaction between the electrode and the reactant may be direct or mediated by redox species. The electrolysis is termed as direct in the former instance but described as indirect in the latter case [60]. The electrochemical transformation of disaccharides has been investigated in two different oxidation methods that use rare metal electrodes.

The most widely studied and promising mediator for the indirect electrochemical oxidation of saccharides is 2,2,6,6-tetramethylpiperidinyloxyl (TEMPO). It has been effectively applied for the transformation of mono-, di-, and polysaccharides. When applied to disaccharides, TEMPO can provide triacids and selectively oxidise primary alcohols. Lactose was quantitatively consumed in these reaction conditions, although the reaction’s selectivity was low. LBA was one of five distinct products that could be identified [56].

Direct electrochemical conversion offers better comprehension of the process and potentially facilitates enhanced control by being able to tune the potential at which the transformation takes place. Although numerous metal electrodes (Au, Pt, Ni, Cu, Co, Ru, Cd, Ir) have been evaluated for the electrochemical oxidation of disaccharides, most of the research primarily focuses on voltammetric analyses of the electrical response of disaccharides on these electrodes [56]. The electrochemical synthesis initiated by Isbell in 1934 yielded an aqueous solution, making it exceedingly challenging to acquire LBA in the solid form. Magariello and Islip (1956) enhanced the process through electrolytic oxidation of lactose utilising graphite electrodes, conducting electrolysis in an aqueous solution with iodine and bromine catalysts in an alkaline calcium salt in an amount to maintain the pH above about 5.2, thereby minimising the decomposition of unoxidised lactose. They have secured a patent for the manufacturing of substantial amounts of LBA with an efficiency of 98% [19].

Subsequent investigations into LBA electrosynthesis, conducted by Druliolle’s team, were focused on platinum, platinum-modified, and gold electrodes in alkaline media, achieving high selectivity (100%) and recovery (>90%) [15,56,61,62,63]. Afterwards, Kokoh et al. focused on the electrocatalytic oxidation of lactose on Au-colloid nanoparticles immobilised on a carbon felt electrode in a flow reactor, which produced LBA in 91% yield [64]. Table 2 presents significant instances of the electrocatalytic oxidation of lactose to lactobionic acid.

## 4. Biological Methods of Lactobionic Acid Production

Biological methods for obtaining LBA can be divided into microbial fermentation and enzymatic biocatalysis. Biotechnological production of LBA is based on the oxidation of the free aldehyde group in the glucose molecule of lactose to form a carboxyl group. The general mechanism of the reaction includes the formation of lactobiono-ơ-lactone as an intermediate product, which is subsequently hydrolysed to lactobionic acid [15,66]. Methods for biocatalytic production of lactobionic acid are based on microbes and microbial enzymes, each of which has its own advantages and economic and technical problems. Microbial fermentation is cost-effective and easily scalable, but the rate of the reaction is slower. On the other hand, enzymatic biocatalysis is efficient but more expensive. Recent developments in genetic engineering and bioprocess optimisation show great promise. The lactobionic acid production is a good example of blending the microbial and enzymatic approaches in industrial biotechnology. Further efforts in research and development in this field are likely to make the production of LBA sustainable, cost-effective, and economically accessible across multiple industries.

### 4.1. Microbial Production of Lactobionic Acid

Microbial fermentation has been extensively studied using various species of bacteria and fungi. These organisms have been evaluated for their ability to oxidise lactose to LBA in an efficient manner. The process typically takes place in two stages. At first, microorganisms are grown in optimal growth conditions with a substrate necessary for lactobionic acid synthesis. Qualitative analysis of compounds produced by fermentation has been reported in various studies [67,68]. Microbial fermentation is carried out at temperatures ranging from 25 to 50 °C, with the controlled pH conditions or sometimes also the appropriate light intensity and wavelength for optimal production [69,70]. Several of the earliest studies used bacteria of the genus *Pseudomonas* spp. [24], which use the lactose oxidation pathway to produce lactobionic acid. Lactose is converted to lactobion-δ-lactone by a quinoprotein glucose dehydrogenase, which uses flavin adenine dinucleotide as an electron transfer mechanism. Lactonase subsequently hydrolyses lactone to produce lactobionic acid [68]. Researchers have focused on such strains as *Burkholderia cepacia* [71], *Acetobacter orientalis* [72], *Enterobacter cloacae* [73], *Acinetobacter* sp. [74], *Psychrobacter* sp. [74], and *Penicillium chrysogenum* [75].

**Figure 5:**
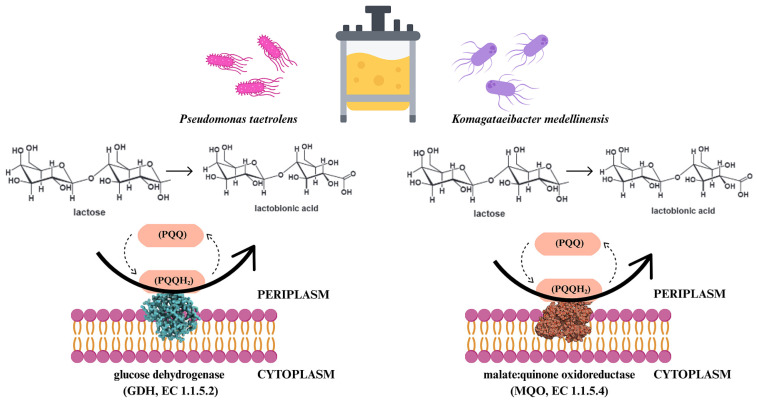
Schematic illustration of the mechanism of the protein-catalysing reaction of malate:quinone oxidoreductase (MQO, EC1.1.5.4) and quinoprotein glucose dehydrogenase (GDH, EC1.1.5.2) from bacteria *Pseudomonas taetrolens* [76,77] and *Komagataeibacter medellinensis* [78]. Lactose oxidation is catalysed by pyrroloquinoline quinone-dependent (PQQ) enzymes. At the same time, pyrroloquinoline quinol (PQQH_2_) is formed from the PQQ cofactor.

Several lactose-oxidising enzymes have been found in bacteria, including the malate:quinone oxidoreductase (MQO, EC 1.1.5.4) from *Pseudomonas taetrolens* [76,77], the quinoprotein glucose dehydrogenase (GDH, EC 1.1.5.2) from *Komagataeibacter medellinensis* [78]. *K. medellinensis* and *P. taetrolens* have Q-GDH, GDH1, and MQO entrenched in the periplasmic side of their inner membranes [74] (Figure 5).

*P. taetrolens* is noteworthy because of the lack of the metabolic efficiency of β-galactosidase activity, which prevents the hydrolysis of lactose to simpler sugars [53,68,79]. Genetic engineering techniques are employed to enhance microbial productivity via the heterologous production of quinoprotein glucose dehydrogenase derived from *P. taetrolens*. Investigations indicate that the expression of the lactose-oxidising enzyme in microorganisms significantly improves their capacity to synthesise lactobionic acid [74,80].

The cost of production is decreased by synthesising lactobionic acid from waste materials. Whey, a by-product of the dairy industry, makes microbial fermentation particularly attractive for industrial applications. Industrial-scale production of LBA using microbial fermentation is well established in the Japanese food industry, where it is used in yoghurts, fermented beverages, and dietary supplements [12].

The use of waste products such as whey is consistent with the principles of zero waste and circular economy, which undoubtedly reduces the production costs of the LBA synthesis process but also has consequences for the way the bioprocess is conducted. There are essentially three types of processes: batch, fed-batch and continuous, which differ in terms of the addition of fresh nutrient and/or the removal of fermented broth during the process. The choice of the appropriate method depends on the requirements of the microorganisms in terms of substrate consumption, product formation and possible inhibitions [81]. In laboratory research, the simplest batch fermentation process remains the most popular, in which all carbon sources and carrier components are added in bulk at the initial stage, and the batch continues to operate until the carbon source is exhausted. In contrast, fed-batch cultivation has become the standard mode of operation adopted by industrial biotechnology to achieve high metabolite yield and productivity. The selection of an appropriate cultivation approach must take physiological information into account, as microorganisms may be exposed to undesirable nutrient depletion or metabolic overload, which in turn leads to an irreversible physiological stress response. In a study conducted by Alonso et al. (2013), different feeding strategies, including co-feeding, continuous feeding, and multi-pulse feeding, were evaluated for the first time for increased production of lactobionic acid from whey by *P. taetrolens* [82].

A summary of current research results of the microbial synthesis of lactobionic acid is provided in Table 3.

### 4.2. Enzymatic Synthesis of Lactobionic Acid

Compared to the microbiological approach, the enzymatic method of LBA production, which uses a crude enzyme initially confirmed by Lin et al. (1993) [89], exhibits a greater yield. However, this process is more complicated and requires controlled synthesis conditions and the participation of a reducing agent. The efficiency and selectivity of oxidoreductases, which can be either oxidases or dehydrogenases, are utilised in enzymatic methods to produce LBA. Molecular oxygen is used by oxidases as an acceptor of hydrogen or electrons. The process by which dehydrogenases oxidise a substrate involves moving hydrogen to an acceptor of either a flavin group or NAD^+^/NADP^+^.

**Figure 6:**
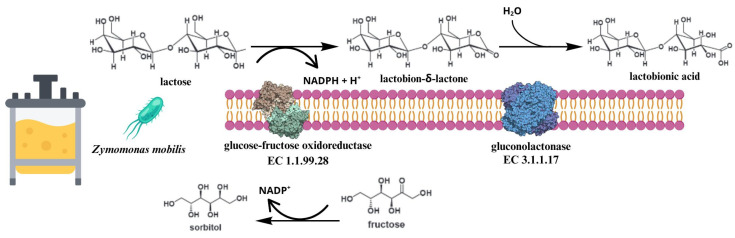
Schematic illustration of the reaction mechanism of the periplasmic GFOR/GL complex of *Z. mobilis*, a species that utilises fructose and glucose as co-substrates natively. The synthesis of LBA and D-sorbitol, respectively, occurs when glucose is replaced with lactose. The enzyme GFOR reduces its cofactor NADPH to NADP while catalysing the oxidation of lactose. This process yields lactobion-δ-lactone, which is then transformed into lactobionic acid by the second enzyme, GL. The second process involves the regeneration of the cofactor in conjunction with D-fructose in order to produce D-sorbitol via GFOR [12,90,91].

Bioconversion of lactose to lactobionic acid by *Zymomonas mobilis* bacteria might suggest that this is microbiological fermentation. However, the application of permeabilised cells in the process indicates that the reaction is purely enzymatic due to the use of the glucose-fructose oxidoreductase (GFOR, EC 1.1.99.28) [90,91] interacting with gluconolactonase (GL, EC 3.1.1.17) in a complex. Substrates and other essential substances can enter the cell through permeabilisation and interact with the enzymes. GFOR, which contains NADP, uses a transhydrogenation process to change different aldose sugars into their corresponding lactones. GL subsequently hydrolyses these lactones to aldonic acids. *Z. mobilis* has GFOR in a soluble form in its periplasm [90] (Figure 6).

The application of this enzyme complex for LBA production, predominantly synthesised by *Z. mobilis*, was initially introduced by Satory et al. (1997) [12,92].

Cellobiose dehydrogenase (CDH, EC 1.1.99.18), quinoprotein glucose dehydrogenase, glucose-fructose oxidoreductase, gluconolactonase, carbohydrate oxidase, pyranose dehydrogenase (PDH, EC 1.1.99.29), carbohydrate acceptor oxidoreductase, malate:quinone oxidoreductase, hexose oxidase, and oligosaccharide oxidase are enzymes that contribute to the synthesis of lactobionic acid [12]. Fungal enzymes are typically released into extracellular space, making them easier to purify and characterise than their bacterial counterparts. As a result, there are more lactose-oxidising enzymes found in fungi than in bacteria [74].

One of the most common enzymes used to synthesise lactobionic acid is cellobiose dehydrogenase. The extracellular glycoprotein cellobiose dehydrogenase secreted by Ascomycota and Basidiomycota fungi oxidises lactose at the C-1 position to yield lactobion-δ-lactone, which spontaneously hydrolyses to LBA. CDH has a heme domain to accept electron transfer from the oxidised to reduced state and a flavin domain with primary catalytic activity [93,94]. Electron acceptors, such as quinones, 2,6-dichloroindophenol (DCIP), 2,2′-azinobis [3-ethylbenzothiazoline-6-sulfonic acid] (ABTS), and metal ions (e.g., Fe^3+^, Cu^3+^, Mn^3+^), increase the efficiency of the reaction by reoxidising the reduced form of the enzyme [17,95,96,97]. 

**Figure 7:**
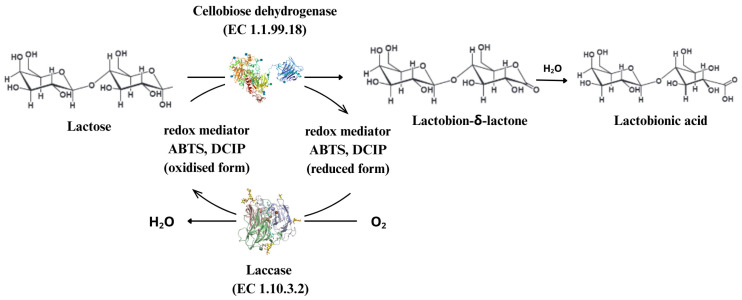
Schematic illustration of cellobiose oxidation using a CDH-ATBS-laccase combination for in situ cofactor regeneration. Oxidation of cellobiose results in the reduction in cellobiose dehydrogenase (CDH), which is reoxidised using 2,2-azinobis-3-ethylbenzthiazoline-6-sulfonic acid (ABTS) or dichloroindophenol (DCIP) as redox mediators. The mediator is regenerated using the enzyme laccase, which is re-oxidised by electron transfer to oxygen [17,95,96,97].

The efficiency of enzymatic production of LBA can be significantly increased by using multi-enzymatic systems [11,12]. For instance, combining CDH with laccase allows continuous regeneration of redox mediators, thus maintaining high reaction rates [29,97,98,99]. The reaction process is illustrated in Figure 7.

Reaction conditions, including temperature, pH, and substrate purity, play a key role in maximising the efficiency of lactobionic acid synthesis. Optimisation of enzymatic processes for LBA extraction remains a challenge due to the high cost of enzyme production and its purification, as well as the need for stable, non-toxic redox mediators [11,12]. A significant advance in the enzymatic production of LBA is the immobilisation of the enzyme. Enzymes bound to a solid support or encapsulated in a polymer are more stable and reusable, which makes the process more economical [66,100]. One of the main advantages of immobilisation, in addition to the financial aspects, is the ease of separation of the products after the reaction [101,102,103]. Currently, the co-immobilisation process, which involves the simultaneous immobilisation of two or more enzymes on the same carrier, is of interest to researchers. As a result, it is possible to efficiently carry out a multi-enzymatic reaction while minimising substrate losses [104,105].

Various methods have been reported to be effective, such as adsorption, covalent bonding, or entrapment in, for example, alginate beads or silica gels [103]. Immobilised cellobiose dehydrogenase has prolonged activity and stability, making it suitable for continuous production systems [106]. 

Biotechnology companies like Novozymes have started producing a mixture of enzymes, LactoYIELD (expresses cellobiose dehydrogenase activity) and Catazyme (catalase (EC 1.11.1.6) that catalyses the decomposition of hydrogen peroxide into water and molecular oxygen), given the technological potential of the enzyme catalysis process [17,107]. Comparative studies of various biocatalytic systems utilising these commercial mixtures, focused on their efficiency in producing lactobionic acid (LBA) from lactose, were conducted by Chidar (2022). These systems were further supplemented with laccase and/or a mediator (ABTS) to achieve the highest possible process efficiency [17]. The investigations on the enzymatic synthesis of LBAs are summarised in Table 4.

Due to their substrate specificity, a number of enzymes can also catalyse the oxidation of lactose to lactobionic acid; nevertheless, it is hard to locate research that has conducted the bioconversion process and evaluated its efficiency. Examples of these biocatalysts comprise hexose oxidase (HOx, D-hexose: O2 1-oxidoreductase, EC 1.1.3.5) from the marine red alga *Chondrus crispus* [108], lactose oxidase (LOD) from *Myrmecridium flexuosum* [109] or pyranose dehydrogenase (PDH, EC 1.1.99.29) from *Agaricus campestris* and *Agaricus xanthoderma* [110].

**Table 4:** Summary of the enzymatic production of LBA.

Lactose-Oxidising Enzyme	Regenerative Enzyme	Redox Mediator	Substrate	Yield (%)	Productivity (g/L/h)	References
GFOR/GL complex *Zymomonas mobilis*	n.d.	n.d.	Lactose	100	5.80	[90]
GFOR/GL complex *Zymomonas mobilis*	n.d.	n.d.	Lactose/ fructose	78	7.6	[91]
CDH from *Athelia* *(Sclerotium) rolfsii*	*Trametes pubescens* laccase	ABTS	Lactose	100	18	[27]
CDH from *Athelia* *(Sclerotium) rolfsii*	*Trametes pubescens* laccase	DCIP	Lactose	100	7	[97]
CDH from *Athelia* *(Sclerotium) rolfsii*	*Trametes pubescens* laccase	ABTS	Lactose	100	19.3	[97]
CDH from *Termitomyces clypeatu*	n.d.	n.d.	Lactose	100	0.01	[111]
CDH from *Myriococcum* *thermophilum*	n.d.	n.d.	Lactose	100	3.3	[112]
CDH from *Pycnoporus* sp.	Laccase	3-Hydroxyanthranilic acid 3-HAA	Lactose	96	3.1	[99]
CDH from *Phanerochaete chrysosporium*	*Cerrena unicolor* laccase	ABTS	Lactose	100	n.d.	[100]
CDH from *Aspergillus fumigatus*	*Trametes* sp. laccase	ABTS	Lactose	100	7.14	[28]
Carbohydrate oxidase from *Microdochium nivale*	Catalase	-	Lactose	98	4.9	[113]
Lactose oxidase (LOX)	Catalase	-	Lactose and whey permeate	98	n.d.	[114]
*Acremonium strictum* glucooligosaccharide oxidase	Catalase	-	Lactose	98	6.7	[115]
Carbohydrate acceptor oxidoreductase from *Paraconiothyrium* sp.	-	-	Lactose	100	14.3	[116]
LactoYIELD	Catazyme	-	Lactose	42–79	7.6–13.2	[17]
LactoYIELD	Catazyme	-	Whey permeate	23–100	11.6–17.5	[17]
LactoYIELD	Catazyme/ laccase	ABTS	Lactose	28.3–100	14.4–21.1	[17]
Malate: quinone oxidoreductase MQO, EC 1.1.5.4	-	-	Lactose	100	0.62	[76]

Note: n.d., not determined.

## 5. Systematic Comparison of Chemical and Biological Approaches

**Figure 8:**
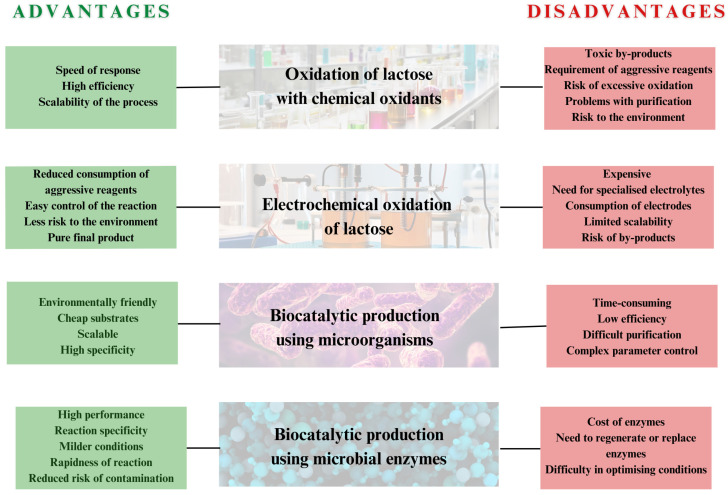
Advantages and disadvantages of chemical and biological methods for obtaining LBA.

Due to the diversity of LBA synthesis methods as well as their respective advantages and disadvantages (Figure 8), this chapter provides a comprehensive comparison based on yield, cost, purity, and environmental impact.

### 5.1. Productivity and Efficiency

The yield and efficiency of lactobionic acid production depend on various aspects, such as substrate utilisation, rate of reaction, and reaction conditions, i.e., pressure, pH, or temperature [16]. The production of LBA in chemical processes displays high reaction rates, making it easily applicable to large-scale industrial production. However, the formation of undesirable by-products necessitates additional purification steps, thereby reducing the overall yield of the synthesis [117,118]. 

On the other hand, biological processes, especially those involving microorganisms, afford specificity in product formation in mild reaction conditions [12]. Although biological methods were considered slow and inefficient compared to chemical methods, genetic manipulations, fermentation optimisations, and immobilisation technologies have greatly improved the efficiency of LBA production [28,119,120]. For *P. taetrolens*, the highest productivity of 26.53 g/L/h was achieved (Table 3). In contrast, the productivity of the enzymatic methods was found to be marginally lower, with a result of 19.3 g/L/h for the CDH from *A. rolfsii* in combination with laccase from *T. versicolor* (Table 4). What is more, immobilisation technologies ensure increased catalyst stability and reusability, and thus efficiency [28,121]. Various studies have reported that immobilised systems have up to 2.4 times higher efficiencies than conventional reactions [28,100], which established biological methods as tough competitors with the chemical methods in terms of productivity.

### 5.2. Costs, Quality, and Purification in the LBA Production Process

The commercial success in the production of LBA depends on three interrelated factors: production costs, the quality, and the efficiency of purification from reaction systems. The following subsection consolidates available scientific information on these key areas.

The cost of production is a major consideration when evaluating methods for producing lactobionic acid on an industrial scale. The global market for lactobionic acid was valued at USD 18.2 million in 2024 and is projected to reach a revised size of USD 22.7 million by 2031, growing at a CAGR of 3.3% during the forecast period [122]. Table 5 shows several producers of lactobionic acid for use in scientific, cosmetic, pharmaceutical, and food applications. The current cost of purchasing LBA varies, depending on the intended use and purity of the product, ranging from USD 80–100 per 25 kg to EUR 372 per 100 g.

Quality has been particularly emphasised, as quality levels significantly influence both the efficiency of production processes and the characteristics of the final products [123]. The effective development of a lactobionic acid (LBA) bioconversion process at an industrial scale necessitates the selection of suitable downstream methodologies for product purification following the completion of LBA bioconversion [124].

The purity and quality of lactobionic acid are of major importance, especially in pharmaceutical and food applications [10,15,122]. Lactobionic acid is produced commercially in three purity categories: 99%, 98%, and 97%. As shown in Table 5, purity grades suggested by producers as well as characteristics such as content of moisture or declared use (e.g., pharmaceutical, food or analytical grade) can vary significantly depending on the market and final purification technology utilised [10,124]. Purification and separation techniques have advanced significantly, with traditional methods such as ethanol precipitation, evaporation, crystallisation, and electrodialysis being supplanted by ion-exchange chromatography, which achieves a 100% yield of LBA, alongside rapid constituent identification via high-performance liquid chromatography (HPLC) [124].

The most standard method of LBA purification and recovery is precipitation with ethanol, which provides a 95–98% pure product sufficient for most scientific applications and cosmetic formulations [71,124]. This is widely accepted in fermentation from whey and dairy media. Moreover, it is technologically not very complex, economically efficient and easily scalable [87]. These others, such as crystallisation, microfiltration, activated carbon adsorption and ion-exchange chromatography, are reserved for products which need to be of higher purity, such as pharmaceuticals or functional foods [11,73].

In general, chemical processes require expensive chemicals and energy-intensive conditions, because high temperatures and harsh oxidising agents are necessary to drive the reaction [34,125,126]. In turn, biological methods, especially those using renewable raw materials, tend to be cost-effective. They often work in milder conditions and therefore use less energy. It should be noted that, although the initial setup costs for bioreactors and fermentation systems are usually higher than for chemical processes, their long-term operating costs are lower due to lower reagent requirements and less need for temperature and pressure limitation [12].

Chemical methods, although frequently able to achieve high yields, generate more unwanted by-products, which must be removed in additional purification steps. This approach is becoming increasingly unattractive, reducing both the quality of the final product and the efficiency of the overall process [127,128,129].

Biological methods have the advantage of providing a much cleaner product in a reaction mixture due to the precision of biological systems, which facilitates better control of reaction conditions and ultimately improves the overall quality of LBA [10,11]. In contrast, the use of immobilised cells or enzymes increases the purity of the product by increasing reaction selectivity [28,130]. Studies have confirmed the excellent performance of processes in which LBA is obtained with biological methods. For example, Baminger’s team used a multi-enzymatic system consisting of cellobiose dehydrogenase and lactase to produce lactobionic acid from lactose with 98% efficiency [27]. As reported by Sulej and her team, biological methods, particularly those using immobilised biocatalysts, can be successfully used to produce high-quality LBA with 93% efficiency of conversion of lactose [29,100].

In a study conducted by Carra’s research team, the integration of bioreactors with immobilised cells of *Z. mobilis* contributed to efficient production of LBA while achieving a high maximum specific production rate of 2.73 mmol/g/h [130].

In addition, immobilisation and co-immobilisation allow reuse of the biocatalyst, which significantly reduces the cost of new enzymes or cells for each process cycle [28,100,131]. Currently, there is no data on the annual production of LBA extracted by biotechnological methods alone. However, assuming that there is a strong emphasis on sustainability, it can be assumed that enzymatic and microbiological methods will slowly begin to displace chemical methods over the course of several years. According to the 2024 report, the rising raw material costs are affecting lactobionic acid production and distribution networks, among other things [122,127]. The use of whey as such or a generally available biological waste would solve this problem [87,130].

The evolution of genetic engineering has contributed immensely to the development of innovative methods for the synthesis of LBA [59,77,80,95]. This new workflow increases the yield of lactobionic acid synthesis with fewer reagents. As a result, the process is simpler, and the production costs are much lower [28,100].

### 5.3. Environmental Impact

Environmental sustainability is steadily assuming greater significance in LBA synthesis applied on a large scale. Other than its technical aspects, it is considered of prime importance while selecting production methods. Although the chemical synthesis of lactobionic acid involving oxidation of lactose with strong oxidants is effective and yields large amounts of product, environmental factors make this method a less attractive option for the following reasons:

(a) The use of non-renewable resources, as many chemical reactants utilised in this process are derived from fossil fuel-based materials [132].
(b) Production of chemical waste—in chemical processes, toxic wastes are produced as by-products; such wastes may need neutralisation prior to disposal [129].
(c) High energy consumption—the high temperatures and pressures needed in the synthesis activity bring about a rise in greenhouse gas emissions, particularly when this energy comes from fossil fuels [45].
(d) The toxicity of reactants—substances used in the process, such as bromine—can pose a risk to ecosystems, especially when waste is improperly handled [117].

Biological production of lactobionic acid based on the use of enzymes or microorganisms is much more environmentally friendly due to the following:

(a) The use of renewable raw materials—the main raw material is lactose, which can be obtained from waste from the dairy industry so as to promote a closed-loop economy [11].
(b) Reduced waste generation—enzymatic and microbial processes produce far fewer by-products, most of which are easily biodegradable [11,12,131].
(c) Low energy consumption—biological reactions occur in mild temperature conditions and low pressure, which results in a decreased carbon footprint [16].
(d) No toxicity—the lack of the need to use toxic chemicals reduces the risk of environmental contamination and adverse effects on human health.

From the sustainability perspective, biological methods are a greener alternative consistent with the idea of minimising the environmental impact caused by industrial activities. The decision of which method to use should take into consideration not only efficiency but also long-term environmental implications and the possibility of integration into circular economy models.

## 6. Conclusions and Future Perspectives

In summary, the comparison of chemical and biological methods for obtaining lactobionic acid shows a significant advantage for biological approaches in a number of key aspects, including higher yields, higher purity of the final product, and elimination of the need for cost-consuming multi-step purification (Figure 8). Although the initial investment in biotechnological production systems may be higher, they prove to be more economical in the long term due to the lower consumption of raw materials and energy, reduced waste, and proven environmental benefits, such as reduced emissions and the elimination of toxic solvents.

A new trend that is becoming increasingly popular over the coming years is the use of genetic engineering for efficient production of LBA, which reduces the cost of its synthesis, waste, and pollution and fits perfectly into the characteristics of green biotechnology-based development. The last 5 years have brought intensive research on co-immobilisation of enzymes used to obtain lactobionic acid. The process increases catalytic efficiency and reduces production costs and waste. Nevertheless, further research is required to optimise these processes and enhance their efficiency and availability on an industrial scale.

## Figures and Tables

**Table 1:** Heterogeneous catalytic oxidation of lactose for lactobionic acid production.

Catalyst	Oxidant	Reaction Conditions	Conversion Yield (%)	Selectivity (%)	Reaction Time (min)	References
Pd/C	O_2_	pH 8, 70 °C	29–97	46–95.8	200	[44,48,49,50]
Pd/Al_2_O_3_	O_2_	pH 9, 100–170 °C	54–99	63–97	200	[51]
Pd/Al_2_O_3_	O_2_	pH 9, 40 °C, flow rate 500 mL min^−1^	100	≥99.5	n.d.	[30]
Pd/SiO_2_	O_2_	pH = 9, 70 °C, stirred at 1300 rpm	16.3–27	30–80	120–200	[22]
Pd-Bi/C	O_2_	pH 7–10, 60 °C	95%	100	60	[44]
Pd-Pb/CaCO_3_	O_2_	pH 8, 70 °C	92	83	200	[49]
Pd-Pt-Bi/C	O_2_	pH 8, 70 °C	93	83	200	[49]
Pt/C	O_2_	pH 8, 70 °C, oxygen flow 3.5 mL min^−1^	87–94	54–75	150	[52]
Pt/Al_2_O_3_	O_2_	pH 9, 100–170 °C, pressures of 100 psig	67–100	0–44	150–300	[23,52]
Pt-Bi/C	O_2_	pH 7, 170 °C	82	71	80–300	[52]
Pt/CeMn	O_2_	pH 9, 100–170 °C	100	95	150–300	[23]
Ru/C	O_2_	pH 8, 70 °C	33–34	17–38	200	[52]
Au/Al_2_O_3_ Au/TiO_2_	O_2_	pH 9, 40 °C, flow rate 500 mL min^−1^	100	≥99.5	n.d.	[30]
Au/Al_2_O_3_	O_2_	pH 9, 65 °C	85–97	100	150–180	[53,54]
Au-Ag/Al_2_O_3_	O_2_	pH 8–11, 65 °C	≥90	100	20–120	[55]

Notes: n.d., not determined.

**Table 2:** Electrocatalytic oxidation of lactose for lactobionic acid production.

Mechanism of Action	Catalyst	Electrode	Reaction Conditions	Conversion Yield (%)	Selectivity (%)	References
Bromine-mediated electrochemical oxidation	Br_2_ (mediator)	Graphite electrodes	pH > 5.2, ≤25 °C, CaCO_3_/NaHCO_3_ buffer	~98	~100	[19,20]
Direct oxidation of primary alcohol groups	Pt	Pt electrodes	Alkaline media, pH 8–11, 25–60 °C	>90	~100	[61,62,63]
Direct oxidation of primary alcohol groups	Au	Au electrodes	Alkaline media, pH 8–11, 25–60 °C	>90	~100	[61,62,63]
Indirect oxidation via hypobromite generation	Electrochemically regenerated Br_2_	Graphite electrodes	pH 5–7, 25–30 °C, electrolysis	80–90	80–90	[65]
Direct electro-oxidation in an aqueous medium (flow reactor) on carbon modified by gold nanoparticles	Au-NMC	Carbon felt electrode with immobilised Au-colloids	Carbonated buffer, pH 10.2	91	98	[56,64]
Indirect electrochemical oxidation	Pt with TEMPO mediator	Glassy carbon anode	Alkaline media NaHCO_3_/Na_2_CO_3_	n.d.	n.d.	[56]

Notes: n.d., not determined.

**Table 3:** Summary of the microbial synthesis of lactobionic acid.

Microorganism	Production Mode/Culture Conditions	Biocatalyst	Substrate	Yield (%)	Productivity (g/L/h)	References
*Burkholderia cepacia*	Shaking flask/110 rpm, 40 °C, 24–48 h	Resting cells	Lactose	100	5.55	[71]
*Pseudomonas taetrolens*	Batch cultivations in stirred tank bioreactor/350 rpm, 30 °C, pH 6.5, 60 h	Whole cell	Whey	100	1.27	[11,53]
*Acetobacter orientalis* *strain* KYG22	Shaking flasks/120 rpm, 28 °C, 72 h	Resting cells	Lactose	99.7	n.d.	[72]
*Pseudomonas taetrolens* (formerly known as *P. graveolens*)	Shaking flasks/25 °C, 165 h	Whole cell	Lactose	75	n.d.	[24]
*Pseudomonas fragi* TCCC11892	Bioreactor/350 rpm, 37 °C, pH 6.5, 36 h	Whole cell	Cheese whey	97	3.3	[83]
*Pseudomonas fragi* NL20W	Bioreactor/200 rpm, 30 °C, pH 6.0, 102 h	Whole cell	Whey powder	100	3.09	[84]
*Pseudomonas putida KT2440* (pBB-GDH1) recombinant strain	Bioreactor/ 25 °C to 40 °C, pH 7.0, 84 h	Whole cell	Cheese whey powder	98.73	2.42	[85]
Recombinant *E. coli* strain with homologous expression of malate quinone oxidoreductase	Batch in flask/PQQ as an electron acceptor, 37 h	Growing cell	Concentrate whey	100	0.62	[77]
*Acinetobacter halotolerans* strain KRICT-1	Bioreactor/30 °C, 7% NaCl	Growing cell	Lactose	n.d.	0.22	[86]
*Komagataeibacter medellinensis (Gluconacetobacter xylinus)* NBRC3288	Batch in a flask	Resting cell	Lactose	n.d.	n.d.	[78]
*Pseudomonas taetrolens* LMG 2336	Stirred tank bioreactor/350 rpm, 30 °C, pH 6.5, 32 h	Growing cell	Sweet whey	100	1.12	[53]
*Pseudomonas taetrolens* LMG 2336	Bioreactor/30 °C, pH 6.5, 48 h	Growing cell	Acid whey	100	1.12	[87]
*Pseudomonas taetrolens* DSM 21104	Bioreactor	Growing cell	Various cheese whey	85.8	26.53	[88]
*Acetobacter* *orientalis*	Bioreactor	Growing cell	Lactose, yeast extract, polypeptone, D-glucose, salts.	98	0.54	[72]
*Enterobacter cloacae* KRICT-1	Batch in flask, 180 rpm, 35 °C, 27 h	Growing cell	Lactose	100	8.72	[73]
*Enterobacter cloacae* KRICT-1	Batch in bioreactor, 40 °C, 27 h	Growing cell	Lactose	100	9.97	[73]
*Pseudomonas taetrolens* LMG 2336 and *Lactobacillus casei* CECT 475	Flask/simultaneous fermentation, 250 rpm, 30 °C	Growing cell	Sweet whey	n.d.	0.02	[79]
*Penicillium chrysogenum*	Shake flask cultures, 120 h	Growing cell	Lactose	50	0.004–0.007	[75]
*Acinetobacter* sp.	Batch in a flask	Growing cell	Lactose	69.5	0.72	[74]
*Acinetobacter* sp. recombinant strain with heterologous expression of quinoprotein glucose dehydrogenase from *Pseudomonas taetrolens*	Batch in a flask	Growing cell	Lactose	97.5	0.94	[74]
*Psychrobacter* sp.	Batch in a flask	Growing cell	Lactose	96.5	1.15	[74]
*Psychrobacter* sp. recombinant strain with heterologous expression of quinoprotein glucose dehydrogenase from *Pseudomonas taetrolens*	Batch in a flask	Growing cell	Lactose	97.3	2.83	[74]

Note: n.d., not determined.

**Table 5:** Some global producers of lactobionic acid (4-O-β-D-Galactopyranosyl-D-gluconic acid) for industrial purposes (V.2025).

Manufacturer/Supplier	Product Description	Cost/Price
**Science research**		
Thermo Scientific Chemicals (Alfa Aesar), Waltham, MA, USA	Purity: 97%, water content: 5.0% max	USD 244 per 100 g
Chem-Impex International, Wood Dale, IL, USA	Purity: ≥98%	USD 60 per 100 g
RPI Research Products International, Mount Prospect, IL, USA	Assay: 97.0–103.0%, water content: ≤5.0%, material source: cow milk	USD 67 per 100 g
Sigma-Aldrich, Merck KGaA, Darmstadt, Germany	Assay: 97% (TLC), solubility 5%, biological source: synthetic	EUR 372 per 100 g
AG Scientific Holdings, LLC, San Diego, CA, USA	Assay: 97%	USD 239–288 per 100 g
BIOSYNTH International, Inc., Gardner, MA, USA	n.d.	USD 302 per 100 g
BOC Sciences Bio-Fermen, Inc., Shirley, NY, USA	Purity: 98%	USD 299 per 500 g
Glentham Life Sciences Ltd., Corsham, UK	Assay: 97.0–103.0%, water content: ≤5.0%	EUR 118 per 100 g
**Cosmetics and Pharmaceutical Intermediates**		
M.C. Biotec Inc., Nantong, China	Assay: 98.0–102.0%, water content: ≤5.0%	USD 40–45 per 100 g
Cavin Resources Malaysia, Miri, Sarawak, Malaysia	Purity: ≥98%, cosmetic/pharmaceutical grade	USD 1000 per 50 kg
**Food Industry**		
Allright GC (Jinan) Biotechnology Ltd., Yiwu, China	Assay: 98.0–102.0%, water content: 1.0% max, medicine grade	USD 80–100 per 25 kg
Shandong WorldSun Biological Technology Co., Ltd., Jinan, China	Purity: 99.9%, food grade	USD 150–200 per 50 kg

Note: n.d., not determined.

## Data Availability

No new data were created or analysed in this study. Data sharing is not applicable to this article.
